# Cardiovascular autonomic dysfunction induced by mechanical insufflation‐exsufflation in Guillain–Barré syndrome

**DOI:** 10.1002/rcr2.1135

**Published:** 2023-04-13

**Authors:** Ryota Kuroiwa, Yoshihisa Tateishi, Taku Oshima, Kazumoto Shibuya, Takeshi Inagaki, Astushi Murata, Satoshi Kuwabara

**Affiliations:** ^1^ Division of Rehabilitation Medicine Chiba University Hospital Chiba Japan; ^2^ Department of Neurology, Graduate School of Medicine Chiba University Chiba Japan; ^3^ Department of Emergency and Critical Care Medicine Chiba Kaihin Municipal Hospital Chiba Japan; ^4^ Department of Emergency and Critical Care Medicine, Graduate School of Medicine Chiba University Chiba Japan

**Keywords:** airway clearance, cardiovascular autonomic dysfunction, complication, Guillain–Barré syndrome, mechanical insufflation‐exsufflation

## Abstract

Mechanical insufflation‐exsufflation (MI‐E) is an effective airway clearance device for impaired cough associated with respiratory muscle weakness caused by neuromuscular disease. Its complications on the respiratory system, such as pneumothorax, are well‐recognized, but the association of the autonomic nervous system dysfunction with MI‐E has never been reported. We herein describe two cases of Guillain–Barré syndrome with cardiovascular autonomic dysfunction during MI‐E: a 22‐year‐old man who developed transient asystole and an 83‐year‐old man who presented with prominent fluctuation of blood pressure. These episodes occurred during the use of MI‐E with abnormal cardiac autonomic testing, such as heart rate variability in both patients. While Guillain–Barré syndrome itself may cause cardiac autonomic dysfunction, MI‐E possibly caused or enhanced the autonomic dysfunction by an alternation of thoracic cavity pressure. The possibility of MI‐E‐related cardiovascular complications should be recognized, and its appropriate monitoring and management are necessary, particularly when used for Guillain–Barré syndrome patients.

## Graphical abstract

We herein describe two cases of Guillain–Barré syndrome with cardiovascular autonomic dysfunction during MI‐E. While Guillain–Barré syndrome itself may cause cardiac autonomic dysfunction, MI‐E possibly caused or enhanced the autonomic dysfunction by an alternation of thoracic cavity pressure. The possibility of MI‐E‐related cardiovascular complications should be recognized, and its appropriate monitoring and management are necessary, particularly when used for Guillain–Barré syndrome patients.

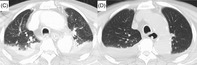

## INTRODUCTION

Guillain–Barré syndrome (GBS) is an immune‐mediated peripheral neuropathy and the most frequent cause of acute flaccid tetraplegia worldwide.^1^ In severe GBS cases, cardiovascular autonomic dysfunctions (such as labile hypertension, bradycardia, and asystole) are frequently observed and may require management in an intensive care unit (ICU). In addition, airway clearance is necessary to prevent respiratory complications because intubation and ventilated management are required in patients with respiratory muscle paralysis. Effective coughing is essential for airway clearance, but it is difficult to clear the airway in patients with respiratory muscle weakness and a decrease in cough reflex. One of the methods for airway clearance is mechanical insufflation‐exsufflation (MI‐E).

MI‐E consists of insufflation of the lungs with positive pressure, followed by an active negative‐pressure exsufflation that creates a peak and sustained flow high enough to provide adequate shear and velocity to loosen and move secretions toward the mouth for suctioning or expectoration. MI‐E devices are useful for respiratory management in GBS. Risks of MI‐E include mechanical injury in the respiratory system, such as pneumothorax,^2^ but to date, there are no reported effects of MI‐E on the autonomic nervous system. This report describes two cases of GBS with severe autonomic dysfunction presumably facilitated by MI‐E.

## CASE REPORT

### Case 1

A 22‐year‐old man was diagnosed with acute motor axonal neuropathy type GBS. He presented with acute motor paralysis of the four extremities and required ventilator support due to respiratory muscle paralysis. He had no history of cardiac or pulmonary disease. After transfer to our hospital, the systolic blood pressure was a maximum 212 mm Hg and minimum 42 mm Hg, with a daily variation of 160 mm Hg. He was admitted to the ICU for cardiovascular management. His heart rate showed sinus tachycardia of 100–120 bpm, but there was no apparent bradycardia. Heart rate variability (HRV) analysis, which evaluates autonomic nervous system function based on changes in electrocardiogram (ECG) R‐R intervals (Memcalc Tonam 16C, GMS, Tokyo, Japan), showed a standard deviation of the normal‐to‐normal intervals (SDNN) of 5.49 msec, low‐frequency (LF) of 14.2 msec^2^, and high‐frequency (HF) of 4.7 msec^2^, all of which were markedly low.

In this case, MI‐E was used for airway clearance because of impaired cough and difficulty with expelling the airway using chest physiotherapy and tracheal suctioning. He experienced transient asystole upon the eighth use of the MI‐E device, but his heart rate immediately recovered without requiring any treatment and there was no hypotension. At the time of the episode's appearance, the insufflation/exsufflation pressure of the MI‐E was +30/−30 cm H_2_O. The use of MI‐E was temporarily discontinued due to safety considerations after this episode. Such episodes occurred not only upon using MI‐E but also tracheobronchial suctioning. The longest observed pause was 6 seconds. A cardiac electrophysiology consultation was requested for multiple episodes of transient asystole, but the results at the time of the 12‐lead ECG did not show any arrhythmias. Transthoracic echocardiography revealed a left ventricular ejection fraction of 55% and a small amount of pericardial effusion, findings that were not suggestive of bradycardia or transient cardiac arrest. However, due to multiple episodes of bradycardia and transient asystole, the decision was made to insert a temporary pacemaker set to VVI mode at 60 beats per minute (bpm).

**FIGURE 1:**
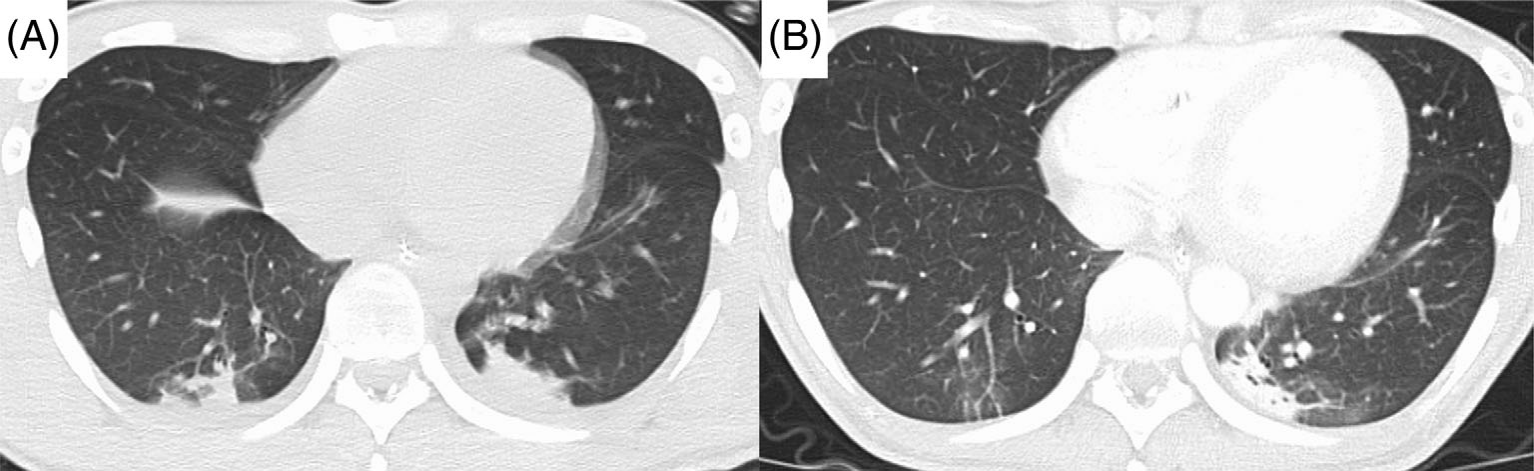
Chest computed tomography of case 1 before (A) and after (B) mechanical insufflation‐exsufflation

After the insertion of a temporary pacemaker, MI‐E therapy was resumed. The heart rate decreased from 107 to 60 bpm and from 103 to 70 bpm on days 5 and 12, respectively, after the insertion of the temporary pacemaker. However, the pacemaker was not activated and the heart rate immediately recovered without any change in blood pressure in both episodes. After these episodes, he had no bradycardia or transient asystole, and the temporary pacemaker was removed 3 weeks after insertion. After the removal of the pacemaker, the patient was able to continue MI‐E without bradyarrhythmia, and airway clearance was maintained for 113 days without any respiratory complications such as marked atelectasis or ventilator‐associated pneumonia (Figure 1). The patient was then transferred to a rehabilitation hospital.

### Case 2

An 83‐year‐old man was admitted to our hospital with progressive acute quadriplegia, respiratory muscle paralysis, and autonomic dysfunction 2 days after the onset of symptoms, and was diagnosed with acute inflammatory demyelinating polyneuropathy type of GBS based on clinical examination and nerve conduction studies. He was admitted to the ICU because of mechanical ventilator support for respiratory failure and the management of blood pressure fluctuation, which ranged from systolic blood pressure of 40–200 mm Hg. HRV analysis showed SDNN 22.6 msec, LF 28.3 msec^2^, and HF 7.7 msec^2^, and this case was also markedly low, suggesting a high degree of autonomic neuropathy.

**FIGURE 2:**
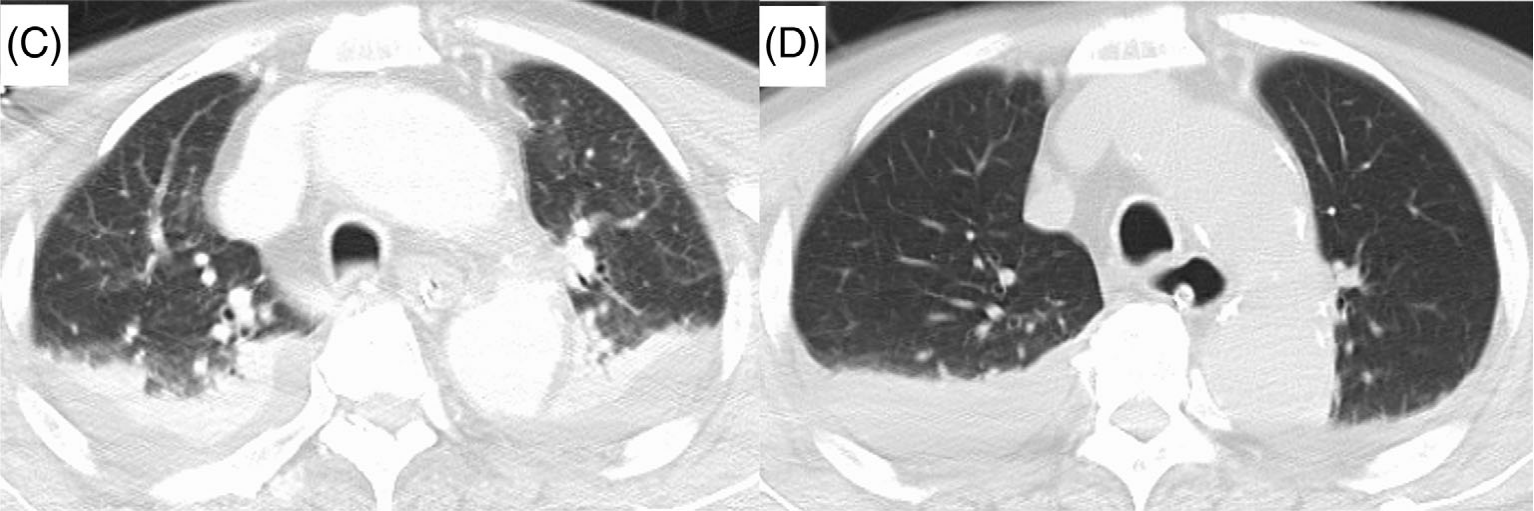
Chest computed tomography of case 2 before (C) and after (D) mechanical insufflation‐exsufflation

In this case, MI‐E was used for airway clearance and improving atelectasis because there was a lot of airway secretion and it was difficult to manage airway clearance using chest physiotherapy and tracheal suctioning. Upon first use of MI‐E, the insufflation/exsufflation pressure was +15/−15 cm H_2_O; a small amount of secretion was expelled, but the systolic blood pressure fluctuated from 164 to 202 mm Hg. The blood pressure fluctuated not only with MI‐E but also with tracheal suctioning, which increased up to 250 mm Hg. MI‐E was performed for three sessions of five cycles as usual treatment, and to take blood pressure fluctuations into account, the normal rest period of 1 min was increased to 5 min, and the subsequent session was performed after confirming that the blood pressure had stabilized. Although the patient experienced severe blood pressure fluctuation, especially during MI‐E and tracheal suctioning the first 5 days of MI‐E use, careful monitoring of blood pressure and appropriate rest during MI‐E reduced blood pressure fluctuation (fluctuation less than +20/−20 mm Hg) and eventually allowed us to increase the MI‐E pressure setting to +30/−20 cm H_2_O. During and after the use of MI‐E, there was no ventilator‐associated pneumonia, atelectasis improved (Figure 2), and airway clearance was maintained. The respiratory function of the patient gradually recovered, and he was weaned from the ventilator 25 days after onset of the disease and was transferred to a rehabilitation center 7 weeks after admission.

## DISCUSSION

This report presents autonomic adverse events in two GBS patients during the use of MI‐E; Case 1 had transient asystole by MI‐E pressure of +30/−30 cm H_2_O, and Case 2 showed fluctuation of blood pressure by MI‐E pressure of +15/−15 cm H_2_O. These adverse events were considered to be associated with cardiovascular autonomic dysfunction because of two reasons. First, both patients had severe GBS and cardiovascular symptoms such as tachycardia and systolic blood pressure fluctuation of more than 85 mm Hg per day. Second, HRV analysis, which evaluates autonomic nervous system function based on changes in ECG RR intervals, showed significantly decreased SDNN, LF, and HF. Previous reports of adverse events with MI‐E use include pneumothorax as a serious adverse event,^2^ and other mild adverse events including dynamic collapse of the upper airways during the exsufflation cycle, gastric distention, premature ventricular contractions, and fatigue. However, the present report is clinically important because there have been no previous reports of autonomic dysfunction associated with the use of MI‐E.

A previous study of cardiovascular complications in autonomic dysfunction related to GBS reported that tachycardia (26.04%), bradycardia (13.54%), labile hypertension (12.5%), and fluctuating heart rate (11.46%) were common among a total of 96 patients.^3^ Only patients that required ventilation developed serious arrhythmias and were often preceded by wide fluctuations in pulse or blood pressure and transient asystole following tracheal suction. The appearance of blood pressure fluctuations and transient asystole associated with the use of MI‐E in this report may be due to afferent fibre lesions of the airway baroreceptors. Ropper et al. postulated that malfunction of afferent baroreceptor reflexes causes labile blood pressure and release of sympathetic efferents leading to catecholamine excess.^4^ This, in turn, sensitizes the left ventricular stretch receptors and other nociceptors, causing compensatory reflex bradycardia. These mechanisms indicate that positive pressure stimulation of the airway by MI‐E stimulated the baroreceptors, which became hypersensitive due to autonomic dysfunction of GBS, and induced blood pressure fluctuations and transient asystole. HRV analysis captures the minute changes in heart rate intervals associated with autonomic regulation, with decreases in SDNN, HF and LF indicating that autonomic dysfunction involves both the sympathetic and parasympathetic nervous system. Both of the cases in this report had lower mean SDNN, LF, and HF values than the GBS patients in Tan et al.^5^ (Table 1), suggesting a higher degree of autonomic dysregulation and a higher risk of circulatory events.

**TABLE 1:** Comparison of the HRV analysis between the present cases and GBS patients in the previous study

	HRV		
	SDNN (msec)	LF (msec^2^)	HF (msec^2^)
Case 1	5.49	14.2	4.7
Case 2	22.6	28.3	7.7
Tan et al. (*n* = 10) Mean ± SD (median)	28.7 ± 7.4	89.5 ± 85.9	17.5 (5.1–65.0)

Abbreviations: HF, high frequency; HRV, heart rate variability; LF, low frequency; SD, standard deviation; SDNN, standard deviation of the normal‐to‐normal intervals.

In all cases, it is important to appropriately assess the risk and intervene at the right time. In Case 1, the patient had repeated bradycardia and sinus arrest not only during MI‐E but also during tracheal suctioning, and the insertion of a temporary pacemaker prevented the development of serious arrhythmias. In Case 2, we successfully minimized blood pressure fluctuations through a modified MI‐E approach (i.e., the cycle of MI‐E and rest). The use of MI‐E is not an absolute contraindication, even in severe cases of autonomic neuropathy such as bradyarrhythmia or the appearance of blood pressure fluctuations; with appropriate monitoring and management, it may be an effective strategy for maintaining airway clearance.

## AUTHOR CONTRIBUTIONS

All authors made substantial contributions to the conception, design, and critical revision of the work for important intellectual content, and gave final approval of the version to be published. In addition, Ryota Kuroiwa contributed to project administration and funding acquisition.

## FUNDING INFORMATION

This study was supported in parts by Japan Society for the Promotion of Science (JSPS) KAKENHI under Grant Number JP 20K19304. The role of the funding body: publication fee was supported by the funding. The funders had no role in study design, decision to publish, or preparation of the manuscript.

## CONFLICT OF INTEREST STATEMENT

None declared.

## ETHICS STATEMENT

The authors declare that appropriate written informed consent was obtained for the publication of this manuscript and accompanying images.

## Data Availability

The data that support the findings of this study are available on request from the corresponding author. The data are not publicly available due to privacy or ethical restrictions.
